# Paroxysmal autonomic instability with dystonia in a patient with tuberculous meningitis: a case report

**DOI:** 10.1186/1752-1947-4-304

**Published:** 2010-09-10

**Authors:** Navin A Ramdhani, Maaike A Sikma, Theo D Witkamp, Arjen JC Slooter, Dylan W de Lange

**Affiliations:** 1 Department of Intensive Care Medicine, University Hospital, Surinam; 2 Department of Intensive Care Medicine, University Medical Center Utrecht, the Netherlands; 3 Department of Radiology, University Medical Center Utrecht, the Netherlands

## Abstract

**Introduction:**

This case report describes an extremely rare combination of paroxysmal autonomic instability with dystonia and tuberculous meningitis. Paroxysmal autonomic instability with dystonia is normally associated with severe traumatic brain injury.

**Case presentation:**

A 69-year-old man of Indonesian descent was initially suspected of having a community-acquired pneumonia, which was seen on chest X-ray and computed tomography of the chest. However, a bronchoscopy showed no abnormalities. He was treated with amoxicillin-clavulanic acid in combination with ciprofloxacin. However, nine days after admission he was disorientated and complained of headache. Neurological examination revealed no further abnormalities. A lumbar puncture revealed no evidence of meningitis. He was then transferred to our hospital. At that time, initial cultures of bronchial fluid for *Mycobacterium tuberculosis *turned positive, as well as polymerase chain reaction for *Mycobacterium tuberculosis*. Later, during his stay in our intensive care unit, he developed periods with hypertension, sinus tachycardia, excessive transpiration, decreased oxygen saturation with tachypnea, pink foamy sputum, and high fever. This constellation of symptoms was accompanied by dystonia in the first days. These episodes lasted approximately 30 minutes and improved after administration of morphine, benzodiazepines or clonidine. Magnetic resonance imaging showed an abnormal signal in the region of the hippocampus, thalamus and the anterior parts of the lentiform nucleus and caudate nucleus.

**Conclusions:**

In patients with (tuberculous) meningitis and episodes of extreme hypertension and fever, paroxysmal autonomic instability with dystonia should be considered.

## Introduction

Fever is a common symptom in the intensive care unit (ICU) and is often related to infectious diseases, either present on admission or nosocomially acquired. Therefore, if a patient develops fever clinicians will obviously be looking for an infection. Hypertension is another phenomenon that is frequently encountered in ICUs and is often attributed to pain or discomfort of the patient. However, both symptoms, alone or in combination with other symptoms, might be caused by infrequent syndromes. Here, we report a patient with attacks of hypertension, fever and autonomous instability caused by paroxysmal autonomic instability with dystonia (PAID). Infection is an extremely rare cause of this syndrome. However, early recognition will prevent over-treatment with antimicrobial agents.

## Case presentation

A 69-year-old man of Indonesian descent was admitted to another hospital with fever and dyspnea. His medical history included tuberculosis in his youth, which was left untreated, ulcerative colitis and a myocardial infarction. His current medication was a beta-blocker, acetylsalicylic acid and azathioprine.

On admission his body temperature was 39.5°C (103°F), and physical and laboratory examination revealed no other abnormalities except for crackles on both sides of the lungs. A chest X-ray showed bilateral nodular changes, which were confirmed by subsequent computed tomography (CT) of the chest. However, a bronchoscopy showed no abnormalities. Cytological examination of sputum was normal. Cultures of blood, sputum, and urine were negative. Auramine, Ziehl-Neelsen stain, and polymerase chain reaction (PCR) for mycobacterium were also negative. The initial screening for tuberculosis was negative and culture results for tuberculosis were not yet available. While waiting for results, anti-microbial treatment was started based upon the suspicion of a community-acquired pneumonia.

The patient was treated with amoxicillin-clavulanic acid in combination with ciprofloxacin. A hypersensitivity pneumonitis caused by azathioprine exposure was also considered and therefore azathioprine was discontinued and prednisone 60 mg a day was started.

However, nine days after admission the patient was somewhat disorientated to time and place and complained of headache. Neurological examination revealed no further abnormalities. A lumbar puncture revealed no evidence of meningitis (Table 1, day 9).

**Table 1:** Laboratory values at admission in the other hospital (day 1) and after transfer to our hospital (day 16).

Measurement	Day 1	Day 9	Day 16	Normal range
Hemoglobin	7.4 mmol/L			8.7-11.1 mmol/L

Leukocyte count	4.7 × 10^9^/L		12 × 10^9^/L	4.0-10.0 × 10^9^/L

Sodium	128 mmol/L			135-145 mmol/L

Potassium	4.0 mmol/L			3.5-5.0 mmol/L

Alkaline phosphatase	107 U/L			

CSF protein		0.42 g/L	1.43 g/L	0.18-0.58 g/L

CSF glucose		2.5 mmol/L	1.8 mmol/L	2.1-3.7 mmol/L

CSF erythrocytes		100/μL	853/μL	None

CSF leukocytes		0-1/μL	82/μL	0-5/μL

CSF granulocytes		-	77%	

CSF lymphocytes		-	23%	

CSF L:E-ratio		1:100	1:10	< 1:100

CSF: cerebrospinal fluid; CSF L:E-ratio: ratio between leukocytes and erythrocytes in the cerebrospinal fluid. Normal values for our hospital are provided in the last column.

**Figure 1:**
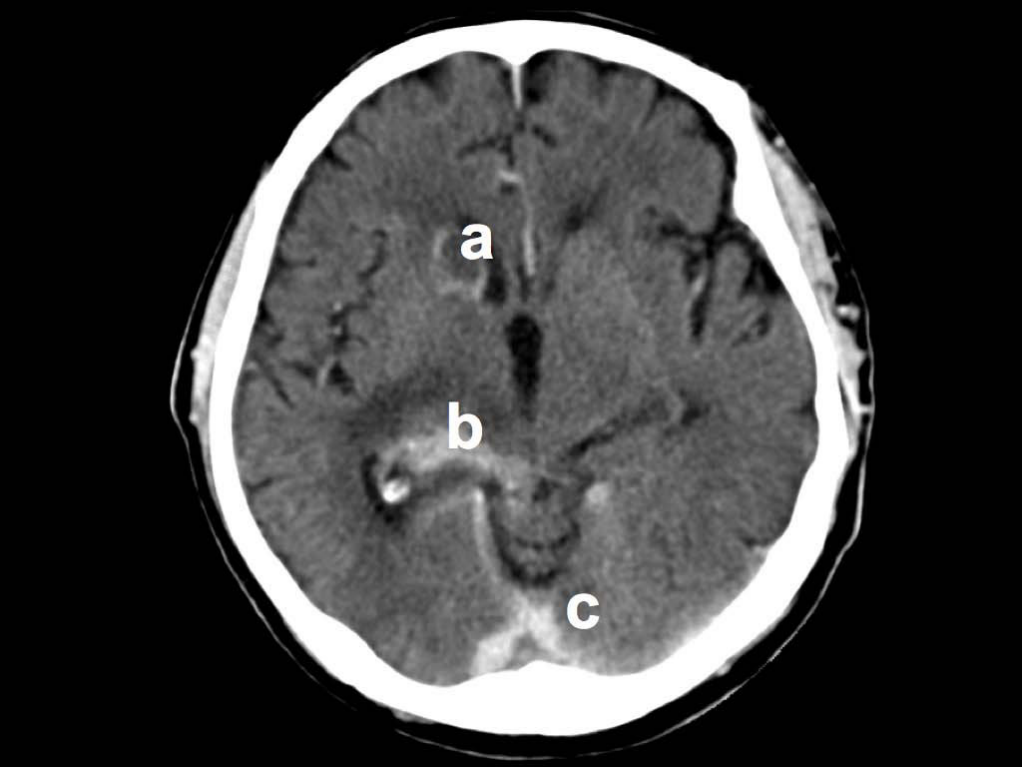
**Axial computed tomography scan of the brain at day nine after presentation**. There are contrast enhancement of (a) the right caudate nucleus, (b) the right medial geniculate nucleus and thalamus, and (c) the cerebellar tentorium. These enhancements are consistent with tuberculomas and leptomeningeal infiltration by *Mycobacterium tuberculosis*.

Nevertheless antibiotics were changed to treat bacterial and viral meningitis. Thereafter, fever and disorientation diminished, but five days later he lost consciousness and developed hemiparalysis. Encephalitis in an immunocompromised host was considered to be the best suitable diagnosis at that time. The patient was referred to our hospital. At that time the initial cultures of bronchial fluid for *Mycobacterium tuberculosis *became positive. Chest radiograph now showed bilateral infiltration of the upper lobes in addition to the previously seen bilateral nodules. Analysis of his cerebrospinal fluid (CSF) corresponded with (myco)bacterial infection (Table 1, day 16). The "opening pressure" of this lumbar puncture was 23 cmH_2_O, corresponding with a moderately raised intra-cranial pressure. The PCR for *M. tuberculosis *was positive in the CSF and subsequent cultures became positive for *M. tuberculosis *as well. We concluded that our patient had disseminated mycobacterial infection, presenting with pneumonia and meningitis. His treatment was changed to isoniazid, rifampicin, pyrazinamid, pyridoxine, moxifloxacin, acyclovir and dexamethasone. He required mechanical ventilation and was transferred to the ICU. An electroencephalogram was performed, which was consistent with diffuse encephalopathy. A subsequent CT scan of the brain showed defects in caudate nucleus, the left occipital lobe and diffusely small lesions of the grey matter, consistent with tuberculomas (see Figure 1).

**Figure 2:**
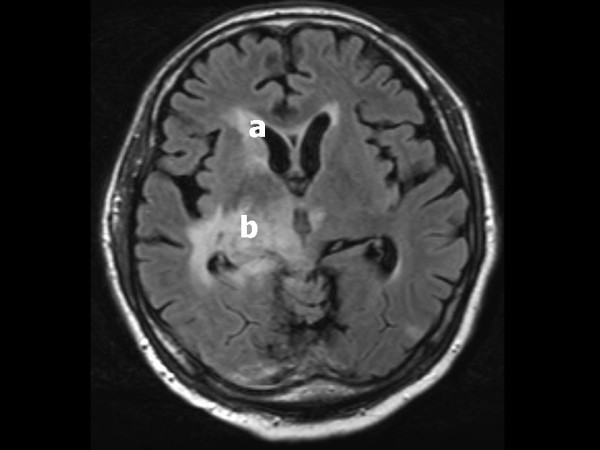
**Axial *T*_2_-weighted magnetic resonance imaging of our patient at day 16 after admission shows an abnormal signal at (a) the corpus callosum and right-sided caudate nucleus as well as (b) the right-sided lentiform nucleus and medial geniculate nucleus extending into the right thalamus and temporal lobe**. These abnormal signals are consistent with tuberculomas and lepto-meningeal infiltration of the thalamic region and the basal nuclei by *Mycobacterium tuberculosis*.

Later, during his stay in our ICU, he developed periods with hypertension (blood pressure 320/250 mmHg), sinus tachycardia (150 beats/minute), excessive transpiration, decreased oxygen saturation (80%) with tachypnea (50/min), pink foamy sputum, and high fever (40°C). This constellation of symptoms was accompanied by dystonia in the first three days. These episodes lasted approximately 30 minutes and improved after administration of morphine, benzodiazepines or clonidine. A drug reaction was considered, but stopping rifampicin did not ameliorate symptoms. PAID due to mycobacterial infection was considered. Magnetic resonance imaging (MRI) showed an abnormal signal in the region of the hippocampus, thalamus and the anterior parts of the lentiform nucleus and caudate nucleus. This was consistent with tuberculous infection (Figure 2). His autonomic instability was treated with beta-blocker and clonidine. A month after admission these periods ceased, which coincided with our patient's improvement. His neurological status improved somewhat (Glasgow Coma Score of E4M5V4) and our patient was discharged to his primary hospital. However, he remained neurologically severely impaired and after three months he died of pneumonia.

## Discussion

This case report is remarkable because PAID is rarely encountered in patients with cerebral infections. Autonomic dysfunction is reported in cases of traumatic brain injury (TBI), hydrocephalus, brain tumors, subarachnoid hemorrhage, and intra-cerebral hemorrhage. The clinical manifestations of autonomic dysfunction are hypertension, a temperature above 38.5°C, a pulse of at least 130 beats per minute, a respiratory rate of at least 40 breaths per minute, intermittent agitation, and diaphoresis. This constellation of symptoms is accompanied by dystonia (rigidity or decerebrate posturing for a duration of at least one cycle per day for at least three days). This can be attributed to altered autonomic activity and recently a new term has been put forth that seeks to more precisely characterize this condition: paroxysmal autonomic instability with dystonia (PAID) [1]. While PAID is predominantly associated with TBI, infection is seldom considered to be a cause of PAID [2].

In 1929 Penfield described a patient with a syndrome called "diencephalic autonomic epilepsy". Signs and symptoms consisted of prodromal restlessness, sudden vasodilatation, sudden rise in blood pressure, lacrimation, diaphoresis, salivation, dilatation or contraction of pupils, sometimes protrusion of eyes, increased rate and pressure of pulse, marked retardation of respiratory rate, elicitation of pilomotor reflex, and rarely loss of consciousness. These features were followed by disappearance of superficial blush and fall of blood pressure, slowing and weakening of pulse, hiccupping, transient shivering, and Cheyne-Stokes respiration. Disturbance of hypothalamic function was considered to be due to a focal epileptic discharge. Autopsy revealed a tumor in the third ventricle [3]. However, later reports of diencephalic seizures did not correlate with electroencephalogram seizure activity, nor were they responsive to anti-convulsants [4-6]. Nowadays the "diencephalic seizures" are considered to constitute a syndrome distinct from PAID [1].

Hereafter, episodic agitation, diaphoresis, hyperthermia, tachycardia, tachypnea, and rigid decerebrate posturing after severe brain injury were first noted in a report by Strich in 1956 [7]. He called these events "brainstem attacks". Subsequently, this constellation of clinical signs has received a variety of labels, including autonomic dysfunction syndrome, dysautonomia, and sympathetic storms [1]. While PAID is noted in approximately 15 to 33% after brain injury, there seems to be no relation with the severity of brain damage. Neuroimaging revealed more frequent evidence of diffuse axonal injury and brainstem injury in those who developed dysautonomia. Often tachycardia, fever and hypertension are the main presenting signs [1]. Our case illustrates this, as extensor posturing was only present in the first days.

The pathophysiology of PAID can be best explained by dysfunction of autonomic centers in the diencephalon (thalamus or hypothalamus) or their connections to cortical, sub-cortical, and brainstem loci that mediate autonomic function, which leads to loss of control of vegetative functions [1,5]. The episodic nature of dysautonomia in PAID might be related to triggering events [8]. Changes in intra-cranial pressure or stimulation of muscle mechanoreceptors and manipulation of endotracheal tube, oropharyngeal suction, and pain may precipitate these attacks [6,9-11]. Indeed, our patient had a slightly raised intra-cranial pressure at the second lumbar puncture. Thermoregulatory dysfunction may also be produced by hypothalamic dysfunction as by the hyper-metabolic state that accompanies sustained muscular contractions. Rigidity and decerebrate posturing are seen experimentally and clinically with lesions in the midbrain, which causes blocking of normal inhibitory signals to pontine and vestibular nuclei [1].

PAID is predominately associated with TBI, but alternative diagnoses should be considered (see Table 2) [10,12].

**Table 2:** Differential diagnosis of hypertension and dystonia.

Neurological
Increased intracranial pressure (ICP)
Non-convulsive epileptic seizures

Central fever

Autonomic dysreflexia

Agitation

Dystonia

Lethal catatonia

Paroxysmal autonomic instability with dystonia (PAID)

**Infectious**

Meningitis

Sepsis

**Drugs/toxins**

Delirium

Serotonin syndrome

Narcotic withdrawal

Neuroleptic syndrome

Malignant hyperthermia

Scorpion envenomation

Gammahydroxybutyrate intoxication

Fenfluramine-phentirmine overdose

**Other**

Pheochromocytoma

Thyroid storm

Renal artery stenosis

Other diseases might share some features with paroxysmal autonomic instability with dystonia or other diseases with hypertension and dystonia.

All these diseases share some features with PAID. Autonomic dysfunction is associated with increased morbidity. While the length of stay in hospital is not different from those without PAID, the length of stay in rehabilitation services is longer. The risk of myocardial infarction and secondary injury due to hemorrhage or elevated intra-cerebral temperature is of concern. Additionally, PAID is also associated with less favorable functional outcomes.

Treatment of PAID is based on case reports and randomized clinical trials are lacking. Next to the treatment of the underlying cause the central sympathetic pathways may be controlled by morphine, bromocriptine [5,9,13,14], clonidine or a non-selective beta-blocker [6,13-15]. Benzodiazepines can also be effective, as in our patient [1,16]. Dantrolene can decrease fever due to prolonged muscle contraction [11,16].

In this case report PAID was secondary to tuberculous meningitis with infiltration of the thalamus hypothalamus parenchyma. Autonomic instability has only been described once in a two-year-old girl with tuberculous meningitis [2]. However, this girl also had hydrocephalus and bilateral hemorrhagic infarctions in her basal ganglia. Conditions that independently have been associated with PAID. Early recognition is of paramount importance to start treatment in an early stage, but recognition is often difficult, as demonstrated in this case.

## Conclusions

PAID is a very rare syndrome in tuberculous meningitis, but recognition and treatment is very important to prevent further complications. In our patient MRI showed abnormalities of the basal nuclei and thalamus-hypothalamus region which may add to previous evidence for the important role of the thalamus in the pathophysiology of PAID.

## Abbreviations

CSF: cerebrospinal fluid; ICU: intensive care unit; PAID: paroxysmal autonomic instability with dystonia; PCR: polymerase chain reaction; TBI: traumatic brain injury.

## Consent

Written informed consent for publication could not be obtained. All reasonable attempts to gain consent from the patient or their next-of-kin have been made. However, every effort has been made to protect patient anonymity and there is no reason to think that the patient or their family would object to publication.

## Competing interests

The authors declare that they have no competing interests.

## Authors' contributions

NAR, MAS, AJCS and DWL treated this patient and analyzed, interpreted the patient data re-garding paroxysmal autonomic instability with dystonia and drafted the manuscript. TDW provided the MRI and CT images and the descriptions. Both AJCS and DWL finalized the manuscript and provided corrections according to their specialty and were major contributors in writing the manuscript. All authors read and approved the final manuscript.
